# CCL2 rs1024611Gene Polymorphism in Philadelphia-Negative Myeloproliferative Neoplasms

**DOI:** 10.3390/genes13030492

**Published:** 2022-03-10

**Authors:** Hossam Hodeib, Dina Abd EL Hai, Mohamed A Tawfik, Alzahraa A. Allam, Amal Selim, Abdallah Ahmed Elsawy, Amira Youssef

**Affiliations:** 1 Clinical Pathology Department, Faculty of Medicine, Tanta University, Tanta 31527, Egypt; hossamhodeib@gmail.com (H.H.); dinaibraheem85@yahoo.com (D.A.E.H.); damirayoussef@yahoo.com (A.Y.); 2 Internal Medicine Department, Faculty of Medicine, Tanta University, Tanta 31527, Egypt; alzahraa.allam@gmail.com (A.A.A.); amalibrahims@hotmail.com (A.S.); abdallahelsawy@hotmail.com (A.A.E.)

**Keywords:** Philadelphia, myeloproliferative, polymorphism, CCL2 rs1024611Gene

## Abstract

Introduction: The onset of the Philadelphia chromosome-negative myeloproliferative neoplasms (MPNs) is caused by acquired somatic mutations in target myeloid genes “driver mutations”. The CCL2 gene is overexpressed by non-Hodgkin lymphomas and multiple solid tumors. Aim of the study: to evaluate the possible association of CCL2 rs1024611 SNP and its expression level and the risk of developing Philadelphia-negative MPNs. Patients and methods: A total of 128 newly diagnosed Philadelphia-negative MPN patient and 141 healthy subjects were evaluated for the genotype distribution of CCL2 rs1024611 and CCL2 expression levels. Results: The CCL2 rs1024611 G/G genotype was more frequent and significantly frequent among PMF and Post-PV/ET-MF patients and the mean CCL2 expression levels were significantly higher in PMF and Post-PV/ET-MF compared to the healthy subjects. The CCL2 rs1024611 SNP was significantly correlated to the CCL2 gene expression level and fibrosis grade. ROC analysis for the CCL2 gene expression level that discriminates MF patients from PV + ET patients revealed a sensitivity of 80.43% and a specificity of 73.17% with an AUC of 0.919 (*p* < 0.001). Conclusion: The CCL2 rs1024611 polymorphism could be an independent risk factor for developing MF (PMF and Post-PV/ET-MF). Moreover, CCL2 gene expression could be potential genetic biomarker of fibrotic progression.

## 1. Introduction

The classical picture of Philadelphia chromosome-negative myeloproliferative neoplasms (MPNs) has been identified as closely related stem cell disorders with overlapping morphologic features, namely, polycythemia vera (PV), essential thrombocythemia (ET), and primary myelofibrosis (PMF). Clonal proliferation of hematopoietic progenitors in the bone marrow, myeloid lineage expansion with variable degree of reticulin/collage fiber deposition, altered peripheral blood cell count, organomegaly, extramedullary hematopoiesis, and increased inflammatory burden are hallmarks of MPNs [1]. PMF either “prefibrotic” or “overtly fibrotic” PMF is the most aggressive phenotype of MPNs. Furthermore, approximately, 15% of ET or PV patients develop a PMF-like phenotype over time, together referred to as post-ET or post-PV MF [2,3,4,5]. Rapidly advancing molecular techniques, and next-generation sequencing technologies (NGS) in particular, have improved our insight about the molecular pathogenesis of MPNs. The landmark discovery of the JAK2V617F mutation changed our concept about the molecular and genetic basis for these disorders [6,7]. Furthermore, our concept about the diagnostic/prognostic criteria of MPNs has changed more and more with the identification of calreticulin (CALR) and Myeloproliferative Leukemia Protein (MPL) mutations [8,9]. Indeed, the onset of MPNs is caused by acquired somatic mutations in target myeloid genes “driver mutations” (JAK2V617F, JAK2 exon 12, CALR, and MPLW515 mutations), and the less common “non-driver mutations” (ASXL1, IDH1/2, EZH2, SRSF2, CBL, LNK, TP53, TET2, etc.) affect MPNs progression [10]. Several studies have highlighted the role of inflammation in the onset and the progression of MPNs; acquired somatic mutations in the neoplastic clone constantly release the inflammatory mediators from activated leukocytes and platelets, and therefore can sustain a chronic inflammatory state [11]. Of note, the JAK/STAT pathway (JAK1, JAK2, STAT3, and STAT5) plays an integral role in the onset of MPNs not only by driving the malignant clone, but also by driving the inflammatory process. Recently, JAK/STAT3-mediated cytokine production from both malignant and nonmalignant cells has been attributed to MPN pathogenesis [12,13]. It is noteworthy that MF patients exhibit higher circulating levels of several pro-inflammatory cytokines such as interleukin-8 (IL-8), interleukin-2 receptor (IL-2R), interleukin-12 (IL-12), interleukin-15 (IL-15), and monocyte chemoattractant protein-1 (MCP-1) in respect to other MPNs and healthy subjects [14,15,16]. The systematic designation for the MCP-1 (subsequently annotated as the rs1024611), a member of the C-C class of the β chemokine family, exerts its effects by engaging to its high-affinity cognate receptor CCR2. CCL2 expression is regulated by series of transcriptional events and leads to CCL2 secretion by a variety of cells, such as monocytes, endothelial cells, fibroblasts, vascular smooth muscle cells, and T cells [17,18,19]. The activation of the CCL2–CCR2 axis triggers immune cell attraction to the site of inflammation and exerts both direct and indirect effects on cancer cells via cancer cell proliferation, stemness, survival, angiogenesis, invasiveness, and metastasis [20]. Moreover, CCL2 is overexpressed by a variety of hematological malignancy (non-Hodgkin lymphomas) and solid tumors as colorectal, pancreatic, gastric, prostate, and breast cancer in particular [21]. Indeed, Single nucleotide polymorphism (SNP) in the regulatory regions of cytokine genes may affect their transcription and may alter their expression levels in that population [22]. In particular, CCL2 rs1024611 SNP (A/G substitution in the regulatory region of the CCL2 gene) alters its transcriptional activity and consequently its expression levels [23]. Recently, the polymorphic allele (G) of CCL2 rs1024611 SNP were more frequent among Post-PV/ET-MF patients and its presence was associated with adverse outcomes [24]. Consistent with these findings, we hypothesized that CCL2 rs1024611 SNP could play a crucial role in the onset and progression of Philadelphia chromosome-negative MPNs. To the best of our knowledge, the available data in the literature ascribing this SNP in Philadelphia-negative MPNs are scarce, insufficient, not clear, and has only been applied to populations with similar ethnic background.

We aimed in the present study to evaluate the possible association of CCL2 rs1024611 SNP and its expression level with the risk of developing Philadelphia-negative MPNs, specifically MF.

## 2. Patients and Methods

This is a cross sectional study carried out from December 2018 to November 2021 in the Hematology/Oncology Unit, Internal medicine Department, Tanta University Hospital, Egypt. A total of 128 newly diagnosed Philadelphia-negative MPN patient (44 PV, 38 ET, 24 PMF, and 22 Post-PV/ET-MF) and 141 healthy subjects of matched age and gender to the patients, as controls were enrolled in the study. The diagnosis of ET, PV, and PMF was based on clinical, laboratory and histopathological data and formulated/classified according to the 2016 WHO classification of myeloid neoplasms [1]. The diagnosis of Post-PV/ET-MF was based on the criteria revised according to WHO and the International Working Group-MRT for Post-PV/ET-MF [4,25]. The molecularly proven driver mutations (JAK2 V617F, CALR, MPL, or BCR-ABL1) were obtained from the patients’ records. Thrombotic and bleeding events were defined as events occurring at the time of diagnosis and/or in the last 2 years preceding diagnosis according to standard definitions. Cardiovascular disease (CVD) risk factors were defined as obesity, smoking, hypertension, diabetes, and dyslipidemia. Splenomegaly was defined as a palpable spleen below the left costal margin.

All patients were eligible for the study after approval by the hospital ethical committee, and the study was performed according to the principles of the declaration of Helsinki; written informed consent was obtained from all patients and control subjects involved in the study.

### 2.1. Peripheral Blood Collection

Whole blood was obtained at the time of Philadelphia-negative MPN diagnosis prior to the initiation of any treatment. Whole blood was collected by means of standard venipuncture in Vacuette Blood Collection Tubes (Greiner Bio-One, Kremsmünster, Austria) containing K2EDTA for complete blood picture and evaluation of peripheral blood film and CCL2 rs1024611 SNP genotyping and containing sodium heparin for isolation of peripheral blood mononuclear cells (PBMCs).

#### 2.1.1. Driver Mutations Detection

##### Real-Time PCR Assay for the Detection of JAK2 V617F Mutation

In brief, 5 μL of genomic DNA was added to 20 μL of the amplification mix according to manufacturer’s instructions (Cat. No.: 673013, QIAGEN GmbH, Hilden, Germany) using the Universal Taqman PCR Master Mix (Applied Biosystem, Foster City, CA, USA). PCR reaction was programmed as follow: 50 °C for 2 min, 95 °C for 10 min followed by 50 cycles of 92 °C for 15 s and 60 °C for 1 min.

##### Real-Time PCR Assay for the Detection of CALR Type 1/Type 2 Mutations

In brief, 5 μL of genomic DNA was added to 20 μL of the amplification mix according to manufacturer’s instructions (Cat. No.: 674023, QIAGEN GmbH, Hilden, Germany) using the Universal Taqman PCR Master Mix (Applied Biosystem). PCR reaction was programmed as follow: 50 °C for 2 min, 95 °C for 10 min followed by 45 cycles of 95 °C for 15 s (denaturation) and 60 °C for 1 min (Annealing/extension) followed by High-resolution melting (HRM) using Rotor-Gene^®^ Q MDx 5plex HRM instrument (QIAGEN GmbH, Hilden, Germany).

##### Real-Time PCR Assay for the Detection of MPL W515L/K Mutations

In brief, 5 μL of genomic DNA was added to 20 μL of the amplification mix according to manufacturer’s instructions (Cat. No.: 676413, QIAGEN GmbH, Hilden, Germany) using the Universal Taqman PCR Master Mix (Applied Biosystem). PCR reaction was programmed as follow: 50 °C for 2 min, 95 °C for 10 min followed by 50 cycles of 95 °C for 15 s and 60 °C for 1 min.

### 2.2. DNA Extraction and Genotyping

Genomic DNA was extracted by using QIAamp DNA Mini Kit (Cat. No.: 51306, QIAGEN GmbH, Hilden, Germany). DNA samples were stored at −20 °C till the time of the assay. DNA samples were genotyped for CCL2 rs1024611 on Applied Biosystems StepOne™Real-Time PCR Systems (Applied Biosystems, Foster City, CA, USA) at molecular biology unit, clinical pathology department, Tanta university hospital, Egypt. In brief, Predesigned TaqMan^®^ SNP Genotyping Assays kit (catalog No. 4351379, Thermo Fisher Scientific, Waltham, MA, USA) for CCL2 rs1024611 was designed to identify the point substitution in the corresponding gene. PCR reaction (Polymerase Chain Reaction) was performed in a total volume of 25 μL. Then, 5 μL of purified DNA was added to a volume of 20 μL of the amplification mix according to manufacturer’s instructions. Thermal profile for PCR was programmed as follow: denaturation at 95 °C for 10 min (Polymerase activation), followed by 40 cycles of 95.5 °C for 15 s (denaturation) and 60 °C for 1 min (Annealing/extension). Data were analyzed and SNPs were determined.

### 2.3. Isolation of Mononuclear Cells, RNA Extraction, and cDNA Synthesis

PBMCs were isolated from heparinized whole blood by using Ficoll density gradient centrifugation. Total RNA was extracted immediately from PBMCs using QIAamp RNA extraction blood Mini kit (Cat. No.: 52304, QIAGEN GmbH, Hilden, Germany) and stored at −80 °C till the time of the assay. The purity and integrity of total RNA were measured using NanoDrop 2000 (Thermo Fisher Scientific, Waltham, MA, USA). Complementary DNA (cDNA) was prepared from RNA using High Capacity cDNA Reverse Transcription Kit (Applied Biosystems, Cat. No. 4368814) according to the protocol provided by the manufacturer’s instructions.

### 2.4. Real-Time Reverse Transcriptase-Polymerase Chain Reaction (RT-PCR)

The expression levels of CCL2 and GAPDH (reference gene) were determined by quantitative RT-PCR by using QuantiTect SYBR Green PCR Master Mix Kit (Cat. No.: 204141, Qiagen GmbH, Hilden, Germany) and PCR reaction was performed on Applied Biosystems StepOne™Real-Time PCR Systems (Applied Biosystems, Foster City, CA, USA) at molecular biology unit, clinical pathology department, Tanta University Hospital, Egypt. In brief, a PCR reaction was performed in a final volume of 20 μL. Then, 1 μL of purified cDNA was added to a volume of 19 μL of the amplification mix according to the protocol provided by the manufacturer’s instructions (9 μL of Master Mix, 0.5 μL of each of the Reverse and the Forward primers (CCl2 or GAPDH) and 9 μL of Nuclease free H_2_O). The following primers were used: CCL2 forward, 5′-CATAGCAGCCACCTTCATTCC-3′ and reverse, 5′-TCTCCTTGGCCACAATGGTC-3′ and GAPDH forward, 5′-CTCCTCCTGTTCGACAGTCAG-3′ and reverse, 5′-CCCAATACGACCAAATCCGTT-3′. Thermal profile for PCR was programmed as follows: 95 °C for 15 min (initial activation), followed by 40 cycles of 94 °C for 15 s (denaturation), 60 °C for 30 s (annealing), and 72 °C for 30 s (extension). All reactions were performed in duplicates. The data were presented as the relative expression of the gene of interest (CCL2) relative to the internal control gene (GAPDH) as determined by the 2 (−ΔΔCT) method.

### 2.5. Statistical Analysis

Data were fed to the computer and analyzed using IBM SPSS software package version 20.0. (Armonk, NY, USA: IBM Corp). The Kolmogorov–Smirnov was used to verify the normality of distribution of variables. Comparisons between groups for categorical variables were assessed using Chi-square test (Fisher or Monte Carlo). ANOVA was used for comparing the different studied groups. Kruskal–Wallis test was used to compare different groups for abnormally distributed quantitative variables and followed by Post Hoc test (Dunn’s for multiple comparisons test) for pairwise comparison. The population of the studied sample was explored to find its equilibrium with Hardy–Weinberg equation. Odds ratio (OR) was used to calculate the ratio of the odds and the 95% Confidence Interval of an event occurring in one risk group to the odds of it occurring in the non-risk group. Receiver operating characteristic curve (ROC) was used to determine the diagnostic/prognostic performance of the markers, an area of more than 50% gives acceptable performance and an area of about 100% is the best performance for the test. Significance of the obtained results was judged at the 5% level.

## 3. Results

**Figure 1:**
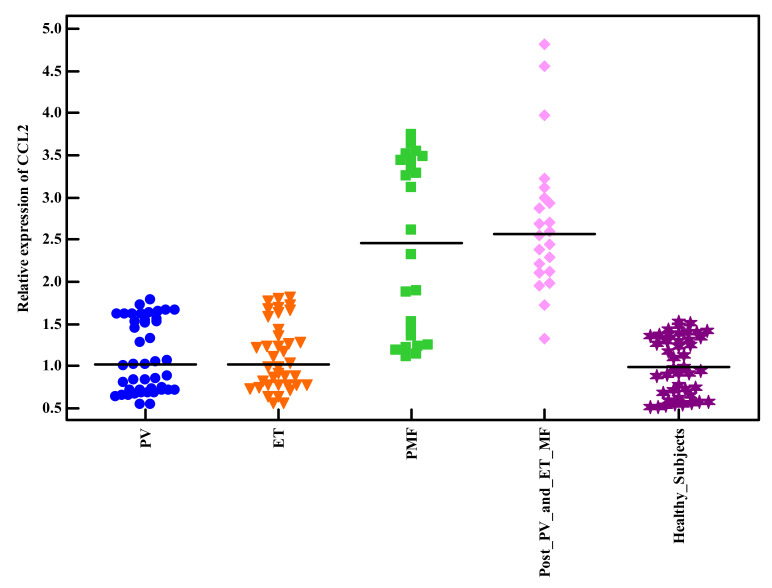
Relative expression of CCL2 among Philadelphia-negative MPNs patients and healthy subjects.

**Figure 2:**
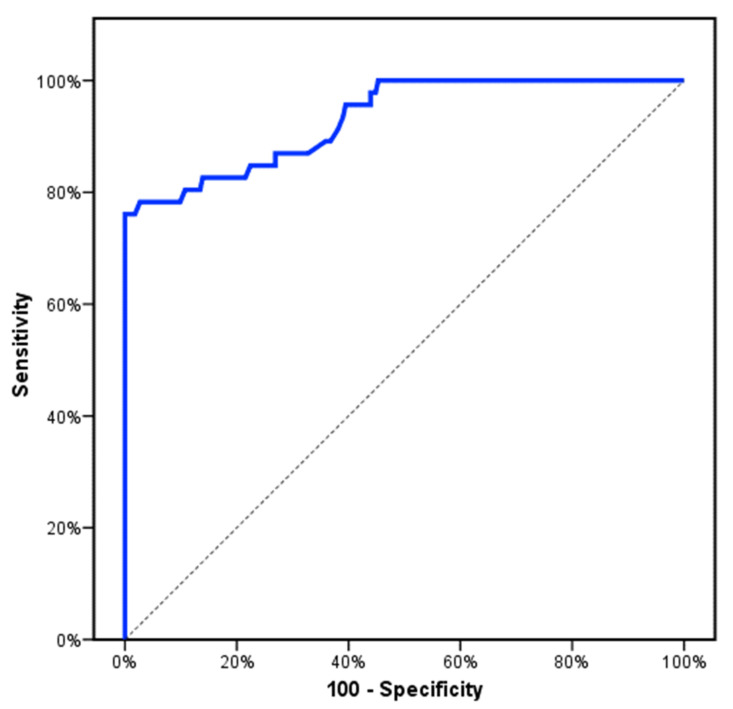
ROC curve for relative expression of CCL2 to discriminate MF patients (PMF and Post-PV/ET-MF) (*n* = 46) from other subjects (*n* = 223).

**Figure 3:**
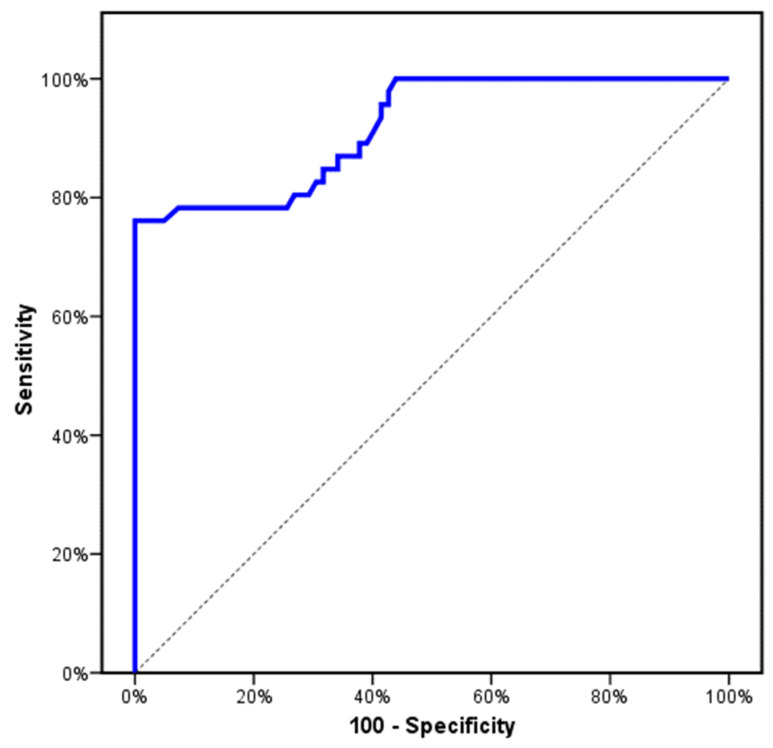
ROC curve for relative expression of CCL2 to discriminate MF patients (PMF and Post-PV/ET-MF) (*n* = 46) from PV and ET (*n* = 82).

In total, 128 patients recently diagnosed as Philadelphia-negative MPNs (44 PV, 38 ET, 24 PMF, and 22 Post-PV/ET-MF) and 141 healthy subjects (control group). There was no significant difference between the studied groups in respect to age and gender (*p* > 0.05). Demographic and baseline characteristic data of the studied groups were summarized in (Table 1). First, we investigated the genotyping and the risk allele frequency of the CCL2 rs1024611 among the studied groups of patients as well as healthy subjects (Table 2). CCL2 rs1024611 polymorphism in both the studied groups of patients and the healthy subjects did not deviate from HWE equilibrium, revealing the reliability of the study samples. The CCL2 rs1024611 AA and A/G genotypes distribution were similar in the studied groups of patients as well as healthy subjects (*p* > 0.05). Interestingly, the CCL2 rs1024611 G/G genotype was more frequent and significantly frequent among PMF and Post-PV/ET-MF patients (*p* = 0.055 and *p* = 0.036), respectively. Additionally, G-allele frequency was higher among PMF and Post-PV/ET-MF patients though it did not reach a significant value (*p* = 0.075 and *p* = 0.061), respectively (Table 2). Next, we evaluated the CCL2 rs1024611 SNP risk associated with the developing of Philadelphia-negative MPNs. Importantly, we observed that the A/G genotype was similarly distributed among the studied groups of patients and the healthy subjects (*p* > 0.05) and patients harboring the AG genotype had a similar risk of the reference group A/A (Table 3). Consequently, we compared the GG genotype vs. A/A and A/G genotypes (the reference group) (recessive genetic model). Surprisingly, patients harboring the GG genotype had high risk of developing PMF and Post-PV/ET-MF (OR = 4.689, 95% CI = 1.19–18.44; *p* = 0.027) and (OR = 5.255, 95% CI = 1.23–22.36; *p* = 0.025), respectively, adjusted by age and gender suggesting that CCL2 rs1024611 polymorphism could be an independent risk factor for developing PMF and Post-PV/ET-MF. Next, we evaluated CCL2 expression levels among the studied groups of patients (at the time of diagnosis) as well as healthy subjects, the mean CCL2 expression levels were significantly higher in PMF and Post-PV/ET-MF compared to the healthy subjects (2.42 ± 1.03 vs. 0.99 ± 0.32) and (2.71 ± 0.86 vs. 0.99 ± 0.32) (*p* < 0.001), respectively (Figure 1). Another interesting finding was made, the mean expression levels of CCL2 in PV, ET, PMF, and Post-PV/ET-MF patients and the healthy subjects were 1.10 ± 0.42, 1.12 ± 0.10, 2.42 ± 1.03, 2.71 ± 0.86, and 0.99 ± 0.32, respectively. In addition, the mean expression levels of CCL2 were higher in Post-PV/ET-MF patients compared to PMF patients though not reached significant value (*p* = 0.257). Interestingly, the CCL2 rs1024611 SNP was significantly correlated to CCL2 gene expression level and fibrosis grade (*p* < 0.001 and *p* = 0.002), respectively. No significant associations were obtained with age, gender, Hb, white blood cell, platelet count, JAK2, CALR, or MPL (*p* > 0.05) (Table 4). In respect to MF patients (PMF and Post-PV/ET-MF), the CCL2 rs1024611 SNP and the relative expression of CCL2 were significantly correlated to IPSS (*p* < 0.001) and no significant associations were obtained with thrombotic events, bleeding events, spleen size, LDH levels, leukemic risk, and overall survival (*p* = 0.610, *p* = 0.618, *p* = 0.636, *p* = 0.129, *p* = 0.698, and *p* = 0.545) and (*p* = 0.356, *p* = 0.667, *p* = 0.889, *p* = 0.803, *p* = 0.526, and *p* = 0.328), respectively. Ultimately, we evaluated the diagnostic/prognostic significance of CCL2 gene expression level; ROC analysis showed that the best cut-off values of CCL2 gene expression level that discriminates MF patients (PMF and Post-PV/ET-MF) from other subjects and from PV + ET patients were >1.36 and >1.52 yielding a sensitivity of 82.61% and 80.43% and a specificity of 78.48% and 73.17% with an AUC of 0.932 and 0.919 (*p* < 0.001), respectively (Figure 2 and Figure 3).

## 4. Discussion

The concept of “onco-inflammation” has been identified by Bottazzi and colleagues to clarify the complex interaction between malignant cells and their inflammatory microenvironment [26]. In solid tumors, the contribution of immune cells, cytokines, and stromal microenvironment in the initiation and the progression of malignancy has been demonstrated; however, in the context of hematological malignancies, it still has not yet been established [27]. Philadelphia-negative MPNs have been considered as typical examples of onco-inflammatory disorders [11,28]. Despite the operational classification of classical MPNs, there are overlapping features in respect to symptoms, laboratory findings, bone marrow morphology, and genetic profile. Furthermore, transformation among disease entities is common; with PV and ET representing the early stage of MPNs, and MF representing the advanced stage of MPNs [27,29]. In the present study we try to introduce CCL2 rs1024611 SNP, a critical molecular player on inflammation, which may have an integral role in the onset and the progression of Philadelphia-negative MPNs from the early stage (PV/ET) to the advanced stage (MF). The main important findings obtained from the present work were that the CCL2 rs1024611 AA and A/G genotypes distribution were similar in the Philadelphia-negative MPNs patients as well as in the healthy subjects, while the G/G genotype was more frequent and significantly frequent among PMF and Post-PV/ET-MF patients. Furthermore, G-allele frequency was higher among PMF and Post-PV/ET-MF patients, though it did not reach a significant value. In addition, patients carrying the GG genotype had high risk of developing PMF and Post-PV/ET-MF compared to the healthy subjects. Similar findings were reported by Masselli et al. [24] who observed no detectable differences in the −2518 A/G SNP (CCL2 rs1024611) genotypic and allelic frequencies of overall MPN, PV, ET, and MF patients vs. control subjects, as well as between single disease entities. Interestingly, patients harboring either a heterozygous or homozygous genotype for the −2518 A/G SNP (A/G + G/G) were significantly more frequent in secondary MF (sMF) vs. PMF. Additionally, the number of A/G + G/G patients was also significantly higher in sMF as compared to controls. Quiet similar findings were obtained by Masselli et al. [30] who reported that PMF male patients were significantly enriched in G/G genotype compared to the PMF female patients and the G-allele frequency was significantly higher as well, while no detectable differences in the genotype distribution and allelic frequencies were found among the PMF- and control-female cohorts. Results obtained from the present study revealed that 10/12 (83.3%) patients carrying G/G genotype were male (*p* = 0.06), this difference could be explained by the small number of patients enrolled in the study. In the present study, we observed that the mean CCL2 expression levels were significantly higher in PMF and Post-PV/ET-MF compared to the healthy subjects and the mean expression levels of CCL2 were higher in Post-PV/ET-MF patients compared to PMF patients. These findings, namely PV and ET patients with relatively low CCL2 expression levels (low inflammatory burden) and PMF and Post-PV/ET-MF patients with high CCL2 expression levels (high inflammatory burden), suggest the functional relevance of this SNP in MF cells (highest chemokine levels) and the inflammatory role of CCL2 gene expression at the onset and development of PMF and Post-PV/ET-MF. Moreover, the CCL2 rs1024611 SNP was significantly correlated to the CCL2 gene expression level and fibrosis grade. In accordance with our findings, Masselli et al. [30] recorded that the G/G genotype significantly overexpressed CCL2 transcript compared to the A/A and the A/G genotypes and the G allele exerts its effect on CCL2 expression in a dose-dependent manner. In agreement with our findings, Wong and colleagues reported that the chemokine gene CCL2 was up-regulated threefold more in overtly fibrotic than in pre-fibrotic MPNs [31]. Of note, CCL2 is up-regulated in other disorders characterized by abnormal fibrosis, such as fibrosing diseases of the liver, lung, and kidney [32]. Importantly, the pattern of inflammatory gene, CCL2 expression did not significantly differ between Philadelphia-negative MPNs (PV and ET) in our study as PV and ET with MF grade 0–1 “prefibrotic” with relatively low CCL2 gene expression, suggesting minimal inflammatory burden compared to PMF and Post-PV/ET-MF with MF grade 2–3 “overtly fibrotic” with the highest CCL2 gene expression, suggesting the highest inflammatory burden. It is noteworthy that several studies performed an analysis of the different cytokine profiles in Philadelphia-negative MPNs. Pardanani et al. [16] observed that high circulating levels of MCP-1, IL-2R, IL-8, and IL-15 were associated with poor anemia response, and that elevated levels of MCP-1, sIL-2R, and IL-15 were clustered with splenomegaly in myelofibrosis (both PMF and post PV/ET MF). Similarly, Cacemiro and colleagues recorded elevated plasma levels of MCP-1, GM-CSF, IFNα, IFN γ, MIP-1α, MIP-1β, RANTES, IP-10, IL-6, IL-10, IL-12, IL-1, IL-4, IL-5, and IL-17 in all three disease entities with the highest level observed in PMF [33]. Pourcelot and colleagues reported elevated levels of MCP-1, IL-4, IL-8, PDGF, and VEGF GM-CSF, and IFNγ, in both PV and ET compared to the controls, and that the levels were significantly higher in ET than PV [34]. This result differs somewhat from those obtained from our study. This discrepancy could be explained by the different laboratory technique (ELISA) that was not performed in the present study. Although our study did not provide transcriptional changes within the bone marrow environment, we recorded inflammatory gene up-regulation in overtly fibrotic MPN. On a separate note, the up-regulated inflammatory genes affect both the neoplastic myeloid clone and the bone marrow inflammatory cells. Indeed, JAK/STAT activity affects clonal myelopoiesis in MPN, and stromal cells in the bone marrow leading to fibroblast activation and overt myelofibrosis [35,36]. Therefore, the present study and the previous studies agree that somatic driver mutations arising in the neoplastic clone represent “bad seed” and the inflammatory microenvironment represents “bad soil” in which clonal selection, expansion, and evolution occur [27,29,37,38].

## 5. Conclusions

CCL2 rs1024611 polymorphism could be an independent risk factor for developing MF (PMF and Post-PV/ET-MF). Moreover, CCL2 gene expression could be potential genetic biomarker of fibrotic progression from early stages of Philadelphia-negative MPNs (PV and ET) to the advanced stage (MF). In addition, CCL2 gene expression could be used as a diagnostic marker for MF (PMF and Post-PV/ET-MF) either alone or in combination with other markers in the context of Philadelphia-negative MPNs. The up-regulation of the CCL2 transcript encoding targetable protein in overtly fibrotic MPN suggests new therapeutic strategies for Philadelphia-negative MPNs patients.

## 6. Limitations of the Study

The present study has several limitations that should be acknowledged. First, the present study involved a relatively small number of Philadelphia-negative MPNs patients. Second, our study did not provide transcriptional changes within the bone marrow environment (in vitro experiment). Ultimately, it is a single center study applied to a population with similar ethnic background.

## Figures and Tables

**Table 1:** Demographic, clinical, and laboratory data of Philadelphia-negative MPNs patients (at time of diagnosis) and healthy subjects.

	PV (*n* = 44)	ET (*n* = 38)	PMF (*n* = 24)	Post-PV/ET-MF (*n* = 22)	Healthy Subjects (*n* = 141)	Test of Sig.	*p*
**Age (years)**							
Mean ± SD.	63.77 ± 6.02	62.03 ± 5.01	63.33 ± 4.78	65.41 ± 4.46	63.26 ± 4.75	F = 1.691	0.152
Median (Min.–Max.)	65 (52–72)	62 (50–71)	64 (55–72)	66 (58–73)	64 (53–72)		
**Gender**							
Male	23 (52.3%)	18 (47.4%)	12 (50.0%)	12 (54.5%)	73 (51.8%)	χ^2^ = 0.373	0.985
Female	21 (47.7%)	20 (52.6%)	12 (50.0%)	10 (45.5%)	68 (48.2%)		
**Hb (g/dL)**							
Mean ± SD.	17.56 ± 0.54	13.95 ± 0.62	10.90 ± 1.92	10.68 ± 1.64	13.56 ± 0.93	F = 238.881 *	<0.001 *
Median	17.30	13.80	11.30	10.55	13.60		
(Min.–Max.)	(17.0–18.60)	(13.0–15.10)	(7.10–13.40)	(6.90–13.10)	(12.10–15.10)		
**WBC count (×10^9^/L)**							
Mean ± SD.	12.14 ± 1.62	8.40 ± 1.17	12.67 ± 6.87	13.05 ± 7.31	7.24 ± 1.77	H = 111.406 *	<0.001 *
Median	12.0	8.30	9.90	10.65	7.30		
(Min.–Max.)	(9.60–14.40)	(6.10–10.30)	(4.40–27.30)	(4.40–29.20)	(4.30–9.80)		
**Platelet count (×10^9^/L)**							
Mean ± SD.	595.6 ± 92.16	812.8 ± 111.5	456.5 ± 229.1	443.5 ± 168.9	284.4 ± 55.84	H = 168.231 *	<0.001 *
Median	591.0	822.0	493.0	477.0	283.0		
(Min.–Max.)	(463–752)	(623–991)	(122–780)	(118–694)	(189.0–392.0)		
**JAK2, *n* (%)**	44 (100%)	21 (55.3%)	16 (66.7%)	19 (86.4%)	NA	χ^2^ = 26.659 *	<0.001 *
**CALR, *n* (%)**	NA	9 (23.7%)	7 (29.2%)	0 (0.0%)	NA	χ^2^ = 20.924 *	MCp < 0.001 *
**MPL, *n* (%)**	NA	4 (10.5%)	3 (12.5%)	0 (0.0%)	NA	χ^2^ = 7.435 *	MCp = 0.024 *
**Triple negative, *n* (%)**	NA	7 (18.4%)	2 (8.3%)	3 (13.6%)	NA	χ^2^ = 9.833	MCp = 0.010 *
**Grading of fibrosis**							
**0–1, *n* (%)**	31 (100.0%)	38 (100.0%)	8 (33.3%)	4 (18.2%)	NA	χ^2^ = 73.673 *	<0.001 *
**≥2, *n* (%)**	0 (0.0%)	0 (0.0%)	16 (66.7%)	18 (81.8%)	NA		
**Thrombotic events**	18 (40.9%)	12 (31.6%)	8 (33.3%)	8 (36.4%)	NA	χ^2^ = 0.858	0.835
**Bleeding events**	1 (2.3%)	1 (2.6%)	3 (12.5%)	3 (13.6%)	NA	χ^2^ = 5.388	MCp= 0.100
**Cardiovascular risk factors**	12 (27.3%)	13 (34.2%)	5 (20.8%)	5 (22.7%)	NA	χ^2^ = 1.650	0.648
**Time evolution of MNPs (years)**	NA	NA	NA	7.41 ± 2.26	NA		
**Spleen size (cm)**							
Mean ± SD.	3.3 ± 4.3	1.4 ± 2.2	5.7 ± 5.4	7.4 ± 7.3	0 ± 0	H = 154.030 *	<0.001 *
Median (Min.–Max.)	1 (0–15)	1 (0–10)	4.5 (0–21)	6 (0–24)	0 (0–0)		
**IPSS**							
Low/intermediate-1	NA	NA	9 (37.5%)	7 (31.8%)	NA	χ^2^ = 0.163	0.686
Intermediate-2/high	NA	NA	15 (62.5%)	15 (68.2%)	NA		
**Dacryocytes**	1 (2.3%)	0 (0%)	16 (66.7%)	18 (81.8%)	NA	χ^2^ = 79.762 *	<0.001 *
**Erythroblastosis**	3 (6.8%)	3 (7.9%)	15 (62.5%)	16 (72.7%)	NA	χ^2^ = 52.346 *	<0.001 *
**LDH (U/L)**							
Mean ± SD.	564.2 ± 150.6	503.9 ± 138.7	614.3 ± 225.7	781.4 ± 302.6	182.3 ± 22.6	H = 204.215 *	<0.001 *
Median (Min.–Max.)	517 (371–885)	488 (290–766)	644.5 (254–987)	844 (281–1271)	186 (137–218)		

SD: Standard deviation; χ^2^: Chi square test; MC: Monte Carlo; F: F for One way ANOVA test; H: H for Kruskal–Wallis test; *p*: *p* value for comparing between the studied groups; *: Statistically significant at *p* ≤ 0.05; NA: Not Analyzed.

**Table 2:** Genotyping of CCL2 rs1024611 and risk allele frequency among Philadelphia-negative MPNs patients and healthy subjects.

	PV (*n* = 44)	ET (*n* = 38)	PMF (*n* = 24)	Post-PV/ET-MF (*n* = 22)	Healthy Subjects (*n* = 141)	*p*
**CCL2 rs1024611**						
**AA**	23 (52.3%)	19 (50.0%)	10 (41.7%)	9 (40.9%)	76 (53.9%)	*p*1 = 0.981 *p*2 = 0.899 *p*3 = 0.055 *p*4 = 0.036 *
**AG**	19 (43.2%)	17 (44.7%)	10 (41.7%)	9 (40.9%)	59 (41.8%)	
**GG**	2 (4.5%)	2 (5.3%)	4 (16.7%)	4 (18.2%)	6 (4.3%)	
**HWp0**	0.432	0.465	0.586	0.520	0.189	
**Allele**						*p*1 = 0.857 *p*2 = 0.664 *p*3 = 0.075 *p*4 = 0.061
**A**	65 (73.9%)	55 (72.4%)	30 (62.5%)	27 (61.4%)	211 (74.8%)	
**G**	23 (26.1%)	21 (27.6%)	18 (37.5%)	17 (38.6%)	71 (25.2%)	

χ^2^: Chi square test; *: Statistically significant at *p* ≤ 0.05; HWp1: *p* value for Chi square for goodness of fit for Hardy–Weinberg equilibrium; *p*1: *p* value for comparing PV and Healthy Subjects, *p*2: *p* value for comparing ET and Healthy Subjects, *p*3: *p* value for PMF and Healthy Subjects, *p*4: *p* value for comparing Post-PV/ET-MF and Healthy Subjects.

**Table 3:** The CCL2 rs1024611 SNP risk associated with the developing Philadelphia-negative MPNs.

	PV vs. Healthy Subjects		ET vs. Healthy Subjects		PMF vs. Healthy Subject		Post-PV/ET-MF vs. Healthy Subjects	
	OR (95% CI)	*p*	OR (95% CI)	*p*	OR (95% CI)	*p*	OR (95% CI)	*p*
**CCL2 rs1024611**								
**AA**	Ref.		Ref.		Ref.		Ref.	
**AG**	1.064 (0.53–2.14)	0.861	1.153 (0.55–2.41)	0.706	1.288 (0.50–3.30)	0.598	1.288 (0.48–3.45)	0.614
**GG**	1.101 (0.21–5.83)	0.910	1.333 (0.25–7.14)	0.737	5.067 (1.22–21.1)	0.026 *	5.630 (1.33–23.80)	0.019 *
**GG vs. AA + AG**	1.071 (0.21–5.51)	0.934	1.250 (0.24–6.46)	0.790	4.500 (1.17–17.35)	0.029 *	5.0 (1.29–19.43)	0.020 *
**Allele**								
**A**	Ref.		Ref.		Ref.		Ref.	
**G**	1.052 (0.61–1.82)	0.857	1.135 (0.64–2.0)	0.664	1.783 (0.94–3.39)	0.078	1.761 (0.90–3.46)	0.100

OR: Odds ratio; CI: Confidence interval; *: Statistically significant at *p* ≤ 0.05.

**Table 4:** Genotype–phenotype correlations in Philadelphia-negative MPNs patients (*n* = 128).

	CCL2 rs1024611			Test of Sig.	*p*
	AA (*n* = 61)	AG (*n* = 55)	GG (*n* = 12)		
**Age (years)**					
Mean ± SD.	63.51 ± 5.02	63.22 ± 5.70	64.25 ± 5.40	F = 0.189	0.828
Median (Min.–Max.)	64.0 (52.0–72.0)	64.0 (50.0–73.0)	65.50 (55.0–72.0)		
**Gender**					
Male	29 (47.5%)	26 (47.3%)	10 (83.3%)	χ^2^ = 5.615	0.060
Female	32 (52.5%)	29 (52.7%)	2 (16.7%)		
**Hb (g/dL)**					
Mean ± SD.	14.27 ± 3.0	14.11 ± 3.17	12.82 ± 2.94	F = 1.129	0.327
Median (Min.–Max.)	14.60 (7.70–18.50)	13.80 (6.90–18.60)	12.20 (9.70–18.40)		
**WBC count (×10^9^/L)**					
Mean ± SD.	11.23 ± 4.33	11.25 ± 4.80	11.69 ± 6.49	H = 0.202	0.904
Median (Min.–Max.)	10.30 (4.90–29.20)	10.20 (4.40–27.30)	9.65 (5.10–27.30)		
**Platelet count (×10^9^/L)**					
Mean ± SD.	621.7 ± 201.1	607.3 ± 203.6	539.8 ± 242.4	H = 1.290	0.525
Median (Min.–Max.)	635 (118–982)	631 (122–977)	518 (148–991)		
**JAK2, *n* (%)**	49 (80.3%)	41 (74.5%)	10 (83.3%)	χ^2^ = 0.776	0.678
**CALR, *n* (%)**	8 (13.1%)	6 (10.9%)	2 (16.7%)	χ^2^ = 0.339	0.844
**MPL, *n* (%)**	4 (6.6%)	3 (5.5%)	0 (0.0%)	χ^2^ = 0.362	MCp = 1.000
**Triple negative, *n* (%)**	4 (6.6%)	7 (12.7%)	1 (8.3%)	χ^2^ = 1.313	0.519
**Grading of fibrosis**	(*n* = 49)	(*n* = 54)	(*n* = 12)		
**0–1, *n* (%)**	41 (83.7%)	36 (66.7%)	4 (33.3%)	χ^2^ = 12.424 *	0.002 *
**≥2, *n* (%)**	8 (16.3%)	18 (33.3%)	8 (66.7%)		
**Relative expression of CCL2**					
Mean ± SD.	1.10 ± 0.49	1.88 ± 0.84	3.16 ± 1.12	H = 47.665 *	<0.001 *
Median (Min.–Max.)	0.91 (0.55–2.39)	1.63 (0.56–3.48)	3.54 (1.72–4.81)		
**Thrombotic events**	19 (31.1%)	21 (38.2%)	6 (50%)	χ^2^ = 1.759	0.415
**Bleeding events**	3 (4.9%)	3 (5.5%)	2 (16.7%)	χ^2^ = 2.513	MCp = 0.280
**Cardiovascular risk factors**	16 (26.2%)	17 (30.9%)	2 (16.7%)	χ^2^ = 1.079	0.583
**Time evolution of MNPs (years)**					
Mean ± SD.	8.56 ± 1.51	7.33 ± 2.60	5 ± 0	H = 11.166 *	0.004 *
Median (Min.–Max.)	9 (5–10)	6 (6–14)	5 (5–5)		
**Spleen size (cm)**					
Mean ± SD.	3.61 ± 5.45	3.95 ± 4.60	5.17 ± 6.19	H = 0.996	0.608
Median (Min.–Max.)	1 (0–24)	2 (0–21)	2 (0–18)		
**IPSS**					
Low/intermediate-1	15 (78.9%)	1 (5.3%)	0 (0%)	χ^2^ = 27.903 *	<0.001 *
Intermediate-2/high	4 (21.1%)	18 (94.7%)	8 (100%)		
**Dacryocytes**	8 (13.1%)	18 (32.7%)	9 (75%)	χ^2^ = 20.737 *	<0.001 *
**Erythroblastosis**	5 (8.2%)	23 (41.8%)	9 (75%)	χ^2^ = 29.599 *	<0.001 *
**LDH (U/L)**					
Mean ± SD.	585.36 ± 206.41	611.13 ± 225.57	548.83 ± 226.16	H = 0.844	0.656
Median (Min.–Max.)	514 (256–1271)	602 (290–1266)	555 (254–1010)		

SD: Standard deviation; H: H for Kruskal–Wallis test; χ^2^: Chi square test; F: F for One way ANOVA test; MC: Monte Carlo; *: Statistically significant at *p* ≤ 0.05; *p*: *p* value for comparing between the studied categories.
